# Maspin differential expression patterns as a potential marker for targeted screening of esophageal adenocarcinoma/gastroesophageal junction adenocarcinoma

**DOI:** 10.1371/journal.pone.0215089

**Published:** 2019-04-19

**Authors:** Sijana H. Dzinic, Zaid Mahdi, M. Margarida Bernardo, Semir Vranic, Haya Beydoun, Nadine Nahra, Amra Alijagic, Deanna Harajli, Aaron Pang, Dan M. Saliganan, Abid M. Rahman, Faruk Skenderi, Berisa Hasanbegovic, Gregory Dyson, Rafic Beydoun, Shijie Sheng

**Affiliations:** 1 Department of Oncology, Wayne State University School of Medicine, Detroit, United States of America; 2 Department of Pathology, Wayne State University School of Medicine, Detroit, United States of America; 3 Tumor Biology and Microenvironment Program of the Barbara Ann Karmanos Cancer Institute, Wayne State University School of Medicine, Detroit, United States of America; 4 College of Medicine, Qatar University, Doha, Qatar; 5 Department of Pathology, University Clinical Center, Sarajevo, Bosnia and Herzegovina; 6 Department of Oncology, University Clinical Center, Sarajevo, Bosnia and Herzegovina; Southern Illinois University School of Medicine, UNITED STATES

## Abstract

**Aim:**

Barrett’s esophagus (BE) is a predisposing factor of esophageal adenocarcinoma/gastroesophageal junction adenocarcinoma (ECA/GEJ Aca). BE patients are stratified and subsequently monitored according to the risk of malignant progression by the combination of endoscopy and biopsy. This study is to evaluate the maspin expression patterns as early diagnostic markers of malignancy in BE patients.

**Materials and methods:**

Immunohistochemistry (IHC) staining was performed on 62 archival core biopsies from 35 patients, including BE without dysplasia (intestinal metaplasia, IM), BE with low grade dysplasia, BE with high grade dysplasia, carcinoma *in situ*, and well to poorly differentiated ECA/GEJ Aca (PD-ECA/GEJ Aca). The intensity and the subcellular distribution of immunoreactivity were evaluated microscopically. Statistical analysis was performed using the χ^2^ and Fisher exact tests.

**Results:**

The level of epithelial-specific tumor suppressor maspin protein inversely correlated with the progression from IM to PD-ECA/GEJ Aca. Lesions of each pathological grade could be divided into subtypes that exhibited distinct maspin subcellular distribution patterns, including nuclear only (Nuc), combined nuclear and cytoplasmic (Nuc+Cyt), cytoplasmic only (Cyt) and overall negligible (Neg). The Cyt subtype, which was minor in both IM and dysplasia (approximately 10%), was predominant in ECA/GEJ Aca as early as well-differentiated lesions (more than 50%: *p* = 0.0092). In comparison, nuclear staining of the tumor suppressor TP53 was heterogeneous in dysplasia, and did not correlate with the differentiation grades of ECA/GEJ Aca.

**Conclusion:**

The Cyt subtype of maspin expression pattern in core biopsies of BE patients may serve as a molecular marker for early diagnosis of ECA/GEJ Aca.

## Introduction

The incidence of esophageal adenocarcinoma/gastroesophageal junction adenocarcinoma (ECA/GEJ Aca) has rapidly increased during the last few decades worldwide [1] and is one of the main causes of cancer mortality [2]. The increased incidence of ECA/GEJ Aca is due, in part, to the prevalence of Barrett’s esophagus (BE) in patients of chronic gastroesophageal reflux disease (GERD). There is evidence to support the disease progression sequence of GERD → Barrett’s esophagus (BE) → dysplasia → ECA/GEJ Aca [3–6].

Detection of early stage gastroesophageal tumors is believed to increase patients’ survival. Currently, diagnosis of BE require endoscopic identification of columnar mucosa extending above the GEJ, plus the presence of intestinal metaplasia (IM) with goblet cells [5,7,8]. The management of BE can vary greatly depending on the presence and severity of dysplasia [9]. Although the overall risk of progression of BE to ECA/GEJ Aca is low (2.7% in patients without dysplasia and around 6% in patients with low grade dysplasia (LGD)), the risk increases to 25% in patients with high-grade dysplasia (HGD). Thus, diagnosis of dysplasia plays an important part in patient management and endoscopic surveillance intervals. According to the American Society for Gastrointestinal Endoscopy, in patients with LGD on biopsy after confirmation by a second pathologist, surveillance endoscopy should be repeated within 3 to 6 months [10–12]. Unfortunately, endoscopy and microscopic examination of tissues are invasive procedures, and there is a concern over an under appreciation of the delayed harms of repeated endoscopy [13]. In addition, in some cases, this nondiscriminatory screening does not lead to the appropriate disease management decisions.

Reliable molecular markers are needed to aid the monitoring of BE progression. A number of genetic and epigenetic changes have been identified as potential markers for ECA/GEJ Aca diagnosis or targeted treatment decisions. For example, less than 20% of ECA/GEJ Aca is Her-2 positive to benefit from Her-2-targeted trastuzumab [14]. Mutations of the tumor suppressor gene *TP53*, rare in IM but prevalent in HGD and ECA/GEJ Aca [3], are associated with a worse prognosis in cancer patients [15,16]. Since loss of *TP53* expression is rare in early stages of disease progression, the potential utility of *TP53* to guide tumor screening is not reliable in these cases. Several other molecules have been shown to correlate with poor prognosis of ECA/GEJ Aca, including transforming growth factor β (TGF-β), urokinase plasminogen activator (uPA), and matrix metalloprotease 1 (MMP1) [17]. To date, only a couple of molecules have been evaluated as potential markers for pre-neoplastic staging of ECA/GEJ Aca progression. For example, CDH17 has been shown to be a sensitive marker of IM. Kallikreins were shown to distinguish dysplastic BE from benign tissue, and correlate with tumor progression [18–20]. It is yet to be determined whether any of these markers can help to stratify prognosis and to reduce the number of unnecessary esophageal-gastroduodenoscopy procedures in BE surveillance screening.

Maspin is an epithelial-specific tumor suppressor gene, the expression of which is commonly downregulated in invasive high-grade cancers [21–24]. A role of maspin in blocking tumor invasion and metastasis has been demonstrated in experimental models of multiple types of cancers [25]. We have shown that deletion of the maspin gene in mice results in embryonic lethality, and conditional maspin knockout leads to context-dependent epithelial pathologies, including hyperplasia of the mammary and glands as well as adenocarcinoma of the lung [26]. Further studies in our laboratory demonstrate that maspin controls the expression of a small set of genes involved in differentiation and in so doing averts stemness [27,28]. The protein amino acid sequence and X-ray crystal structure indicate that maspin may act as a serine protease-like molecule to inhibit serine protease like enzymes [29,30]. To that end, we have shown that maspin acts as an endogenous inhibitor of histone deacetylase 1 (HDAC1), which has a catalytic site similar to that of serine proteases [31].

Despite the absence of signature sequences for specific subcellular destinations, emerging evidence revealed that, in early stages of tumor progression, maspin is also dynamically regulated at the level of subcellular distribution. In the normal epithelium, maspin is predominantly nuclear. The retention of maspin in the cytoplasm correlates with cell transformation and tumor progression in various types of cancers including lung and prostatic Aca [32–35]. Nuclear maspin, as opposed to combined nuclear and cytosolic maspin, correlates with better patient prognosis of early stage lung Aca [36–39]. The current study is the first to examine the association of maspin expression in BE and progression to ECA/GEJ Aca. Our results demonstrate that maspin cytoplasmic subcellular pattern may be a sensitive diagnostic marker for early stages of ECA/GEJ Aca.

## Materials and methods

### Tissue specimens

De-identified, formalin-fixed, paraffin-embedded tissue samples from the archives of the Pathology departments of the Wayne State University and the Sarajevo University Clinical Center (diagnosed from 2006 to 2015) were independently examined by four academic pathologists (R.B., Z.M., S.V. and F.S.). This study was approved by the institutional review board of the Detroit Medical Center/Wayne State University (#064516M1E/DMC#13915). As summarized in **Table 1**, the 35 core biopsy specimens encompassed a total of 70 pathologically diagnosed entities including normal GEJ tissue (N) and BE (IM) without dysplasia, BE with LGD or HGD and ECA/GEJ Aca of different grades (i.e., ECA/GEJ Aca *in situ*, well-differentiated ECA/GEJ Aca (WD-ECA/GEJ Aca), moderately-differentiated ECA/GEJ Aca (MD-ECA/GEJ Aca), and poorly-differentiated Aca (PD-ECA/GEJ Aca).

**Table 1:** Human GEJ tissues.

	Number of cases	
Histological Grade	Clinical Diagnosis	Pathological Grade
N (Gastric)		8
IM	4	17
LGD	2	4
HGD	2	7
Aca *in situ*	2	7
WD-Aca	4	6
MD-Aca	8	8
PD-Aca	13	13

### Immunohistochemistry (IHC)

Formalin-fixed paraffin-embedded tissues were cut into 5 μm thick sections and baked at 65°C for 2 h. The slides were deparaffinized in Histo-Clear (National Diagnostics) and rehydrated through a graded descending series of ethanol concentrations in water (v/v) from 100% to 75%. For IHC staining, heat-induced epitope retrieval was carried out in a decloaking chamber (Biocare Medical) using EDTA buffer (Life Technologies, pH 8.0). The slides were treated with 3% hydrogen peroxide for 30 min to block endogenous peroxidase activity and CAS-Block (Invitrogen) for 10 min to block nonspecific background. The consecutive sections were incubated with monoclonal antibodies against maspin (BD Pharmigen, 554292, 1:100) and against TP53 (Ventana Medical Systems, 760–2542, pre-diluted) overnight at 4°C. Normal mouse IgG was used as an isotype control. Next, sections were washed with PBS and incubated with biotinylated secondary anti-mouse antibody (Vector Laboratory, BA-2000, 1:200) for 30 min. For antigen detection, the slides were first incubated with Vectastain ABC reagents (Vector Laboratories) for 30 min and DAB peroxidase substrate solution (Vector Laboratories) for 3 min. The slides were counterstained with hematoxylin, dehydrated through a graded ascending series of ethanol concentrations in water (v/v) from 75% to 100%, and mounted for microscopic analysis. Maspin IHC stained slides were subjected to histopathological evaluation in a blinded fashion by board-certified surgical gastrointestinal pathologists (R.B., Z.M., and A.M.R.). Specifically, consecutive microscopic fields of each specimen were scanned microscopically to score the staining intensity and subcellular localization of maspin in each field (with up to 500 epithelial cells) as previously described [36]. The patterns of maspin subcellular localization include nuclear only (Nuc), combined nuclear and cytoplasmic (Nuc+Cyt), cytoplasmic only (Cyt), and negligible (Neg).

### Statistical analysis

Associations between each histopathological group and maspin subcellular expression pattern subtypes were evaluated using the χ^2^ test or the Fisher exact test where appropriate. A *p* < 0.05 was considered statistically significant.

## Results

### The complexity of maspin differential expression in ECA/GEJ Aca progression

**Fig 1:**
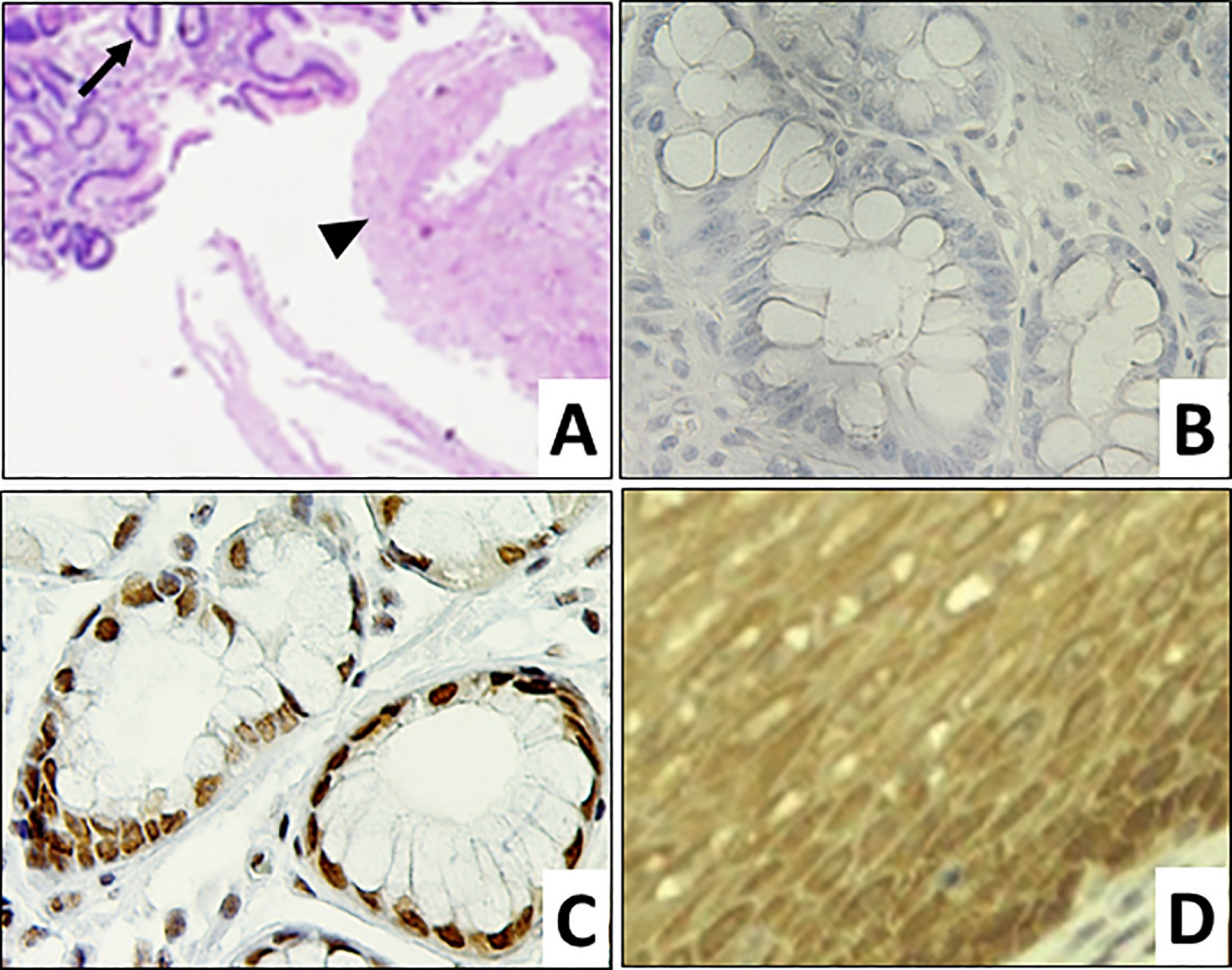
Maspin expression in normal GEJ tissues. Representative microscopic images of normal GEJ tissues. **A.** H&E of squamous (►) and gastric glandular (→) components (100x) of normal GEJ. **B.** Negative control of maspin IHC of gastric glandular epithelium (400x). **C.** Maspin IHC of gastric glandular epithelium (400x). **D.** Maspin IHC of squamous epithelium (400x).

To examine the potential differential expression of maspin in ECA/GEJ Aca progression, we collected 35 core biopsy specimens of GEJ/lower esophagus (LE) from multiple biopsy sites of 12 patients plus single biopsy sites of 8 patients. These specimens encompassed 8 normal GEJ tissues and 62 GEJ/LE lesions of 7 different pathological grades (**Table 1**). Normal GEJ tissues were identified with unremarkable squamous epithelia and gastric glandular epithelia (**Fig 1A**). Using IHC, we found that maspin was expressed at a high level in epithelial cells of the normal GEJ tissues but was undetectable in the stroma (**Fig 1C and 1D**). It was noted that normal gastric glandular tissues expressed maspin mostly in the Nuc pattern (88%) (**Fig 1C**), while a small subtype expressed maspin in the Nuc+Cyt pattern (12%). Normal esophageal squamous epithelial cells uniformly expressed maspin in the Nuc+Cyt pattern (100%) (**Fig 1D**). The negative control, without the primary antibody, confirmed the specificity of the maspin antibody in the IHC staining (**Fig 1B**).

**Fig 2:**
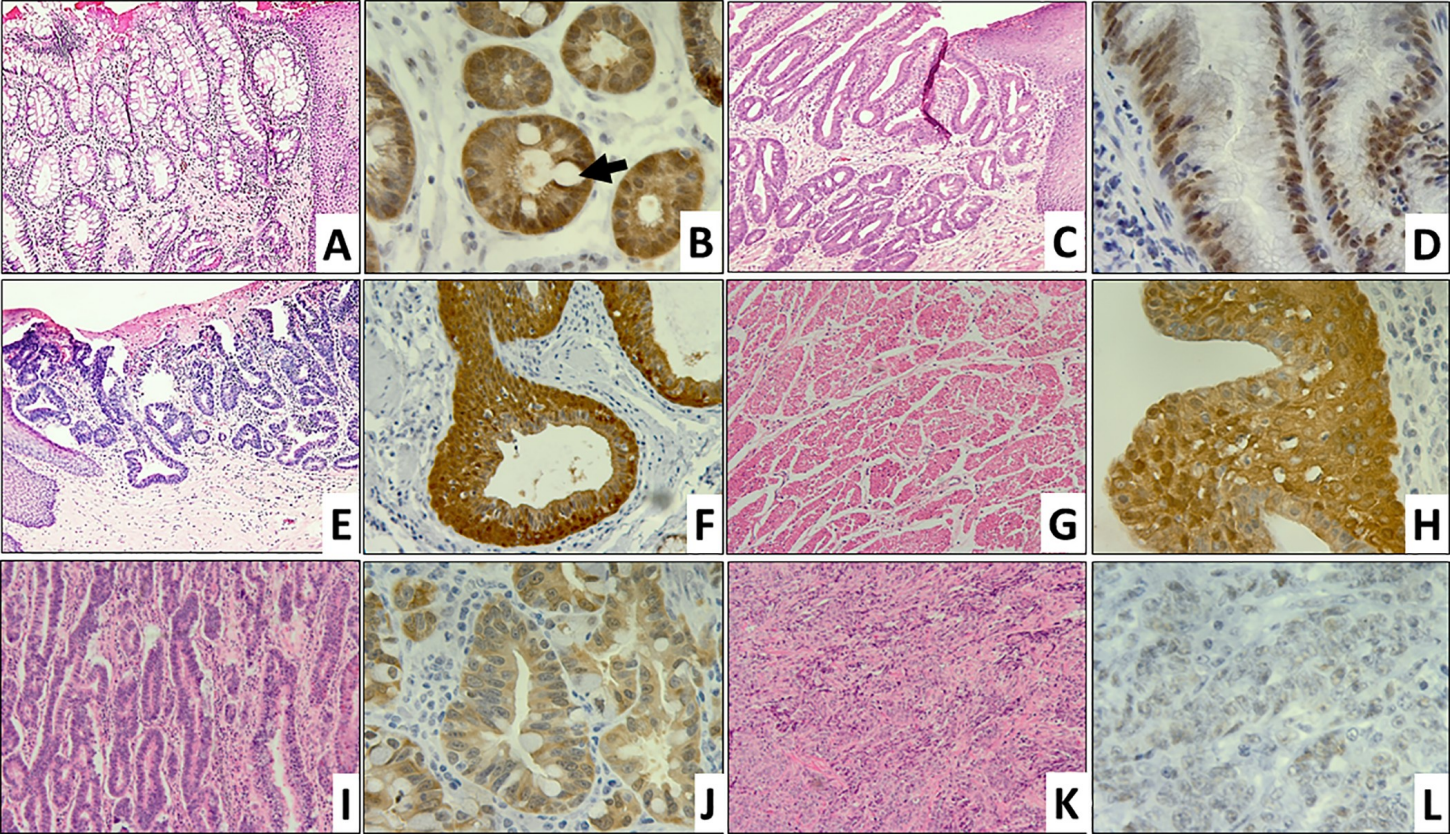
Differential expression of maspin in ECA/GEJ Aca progression. Representative microscopic images of GEJ lesions. H&E (A, C, E, G, I, and K, 100x) and maspin IHC (B, D, F, H, J and L, 400x) staining, respectively, of: **A.** and **B.** IM showing Goblet cells (→); **C.** and **D.** LGD; **E.** and **F.** HGD; **G.** and **H.** WD-ECA/GEJ Aca; **I.** and **J.** MD-ECA/GEJ Aca; and **K.** and **L.** PD-ECA/GEJ Aca.

The clinical BE tissues featured cuboidal epithelial cells of IM and goblet cells. LGD lesions had unremarkable squamous mucosa and atypical glandular cells displaying nucleomegaly and nuclear hyperchromasia that extends to the superficial epithelium. HGD lesions had branching and glandular fusion with remarkable cytological atypia and the absence of stromal desmoplastic or diffuse growth. Transformed cells in ECA/GEJ Aca *in situ* had atypical crowed glands with diffuse growth and non-infiltrating patterns. WD-ECA/GEJ Aca and MD-ECA/GEJ Aca lesions had infiltrative growth patterns with areas of desmoplasia. In addition, PD-ECA/GEJ Aca featured atypical cell sheets. As shown in **Fig 2B**, the cuboidal IM epithelial cells in the BE specimens expressed maspin at a high level. Following the hypothetical tumor progression sequence of IM → LGD → HGD → ECA/GEJ Aca *in situ* → WD-ECA/GEJ Aca → MD-ECA/GEJ Aca → PD-ECA/GEJ Aca, the level of maspin expression, as judged by the intensity of the maspin IHC signal, progressively decreased (**Fig 2D, 2F, 2H, 2J and 2L**).

**Fig 3:**
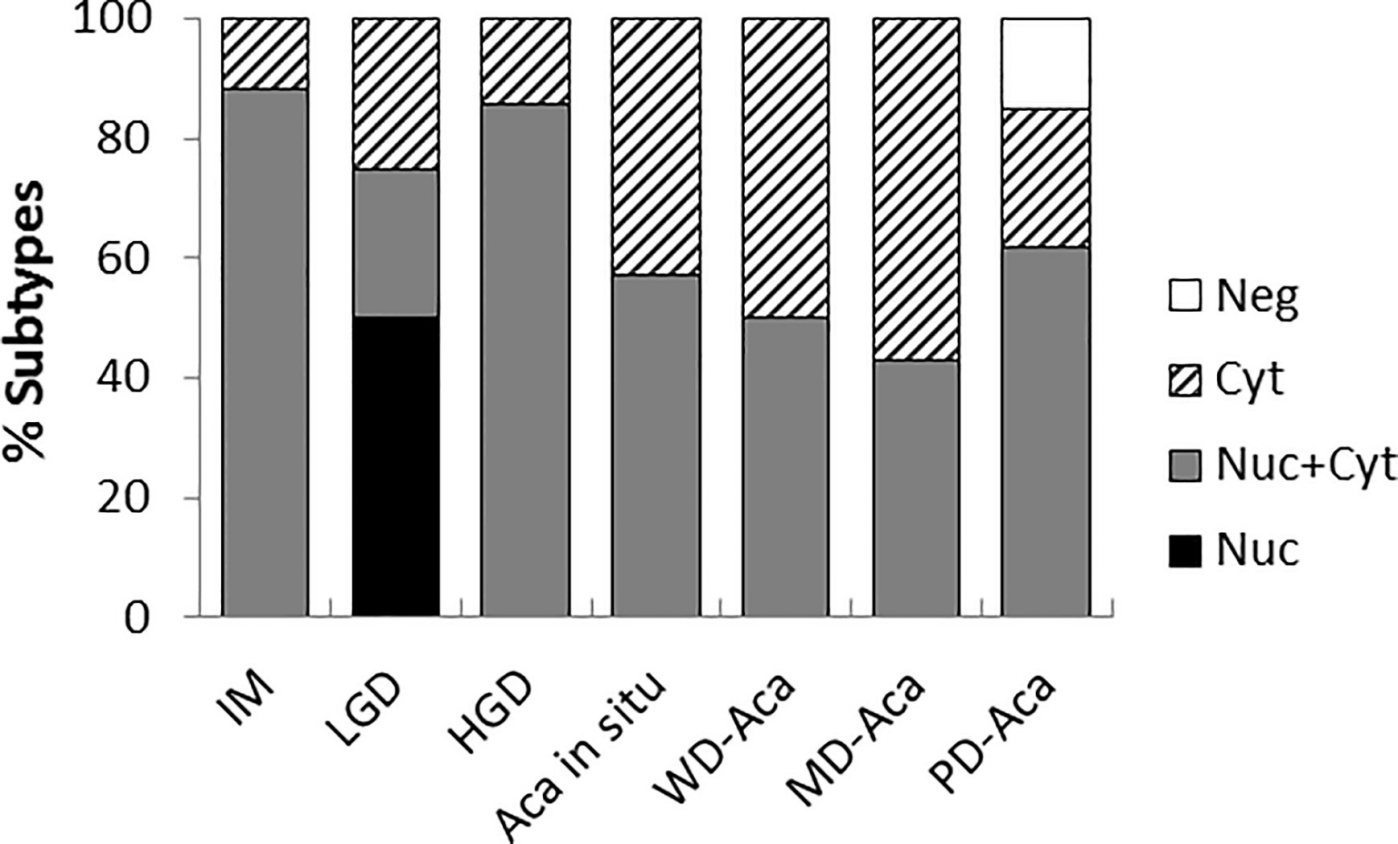
Profiles of maspin expression subtype in different ECA/GEJ pathological diagnosis. The contribution of each subtype is presented as a percentage of the total number of cases of each pathological grade. Nuc: nuclear only; Nuc+Cyt: combined nuclear and cytoplasmic; Cyt: cytosolic only; and Neg: no maspin detection.

In addition, we noted that maspin was differentially expressed at the level of subcellular distribution in ECA/GEJ Aca progression (**Fig 2**). The cuboidal epithelial cells in the IM lesions were divided into two subtypes with maspin in the Nuc+Cyt pattern (88%), and the Cyt pattern (12%) (**Fig 2B**). The LGD lesions were divided into three subtypes with maspin in the Nuc pattern (50%), the Nuc+Cyt pattern (25%), and the Cyt pattern (25%) (**Fig 2D**). The HGD lesions were divided into two subtypes with maspin in the Nuc+Cyt pattern (86%), and the Cyt pattern (14%) (**Fig 2F**). All lesions of ECA/GEJ Aca *in situ*, WD-ECA/GEJ Aca (**Fig 2H**) and MD-ECA/GEJ Aca (**Fig 2J**) were divided into two subtypes, one with maspin in the Nuc+Cyt pattern and the other with maspin in the Cyt pattern. As compared to HGD, more ECA/GEJ Aca lesions had maspin in the Cyt pattern, ranging from 43% to 57%. The PD-ECA/GEJ Aca lesions were divided into three subtypes with maspin in Nuc+Cyt (62%), Cyt (23%) and Neg (15%) patterns (**Fig 2L**). The patterns of maspin expression associated with different lesions, as shown in **Fig 3**, were not dependent on whether the lesions were identified in the same patient or different patients.

To our knowledge, this is the first evidence that maspin expression is epithelial-specific in GEJ. The level of maspin expression inversely correlated with ECA/GEJ Aca progression. Further, each pathological grade of the GEJ lesions could be divided into subtypes based on their distinct maspin subcellular distribution patterns. While IM, HGD, ECA/GEJ Aca *in situ*, WD-ECA/GEJ Aca and MD-ECA/GEJ Aca were composed of two subtypes (i.e., maspin in Nuc+Cyt and Cyt patterns), LGD and PD-ECA/GEJ Aca had a more complex subtype composition, each comprising three subtypes of distinct maspin expression patterns.

### Increased Cyt pattern of maspin expression correlates with the transition to ECA/GEJ Aca

We are aware that the level of maspin expression, as determined by IHC or other methods, may not be easily translated into clinical application for ECA/GEJ Aca diagnosis, at least in part, due to lack of reliable standardization logarithms. It is intriguing to postulate that the more distinctive maspin subcellular distribution patterns may be more easily adapted to aid in ECA/GEJ Aca screening and diagnosis. To explore this possibility, we performed the χ^2^ test to examine whether the GEJ lesion subtype composition, according to the maspin expression patterns, varied as a function of ECA/GEJ Aca progression. As summarized in **Table 2**, the subtype composition of IM was significantly different from that of normal gastric glandular tissues (*p* = 3.2e-5), and was significantly different from that of LGD (*p* = 0.005). However, the subtype composition of IM was not significantly different from those of HGD, ECA/GEJ Aca *in situ*, WD-ECA/GEJ Aca, and PD-ECA/GEJ Aca, respectively (*p* > 0.05). The Fisher exact test was also performed for the same sets of pairs and confirmed the results of the χ^2^ test.

**Table 2:** Statistical analysis of the differential maspin patterns.

Differentiation Grade	Maspin Patterns				Paired Comparison	χ^2^ Test	Fisher Test
	Nuc	Nuc+Cyt	Cyt	Neg		*p*	*p*
**Normal Gastric**	7 (87.5%)	1 (12.5%)	0 (0.0)	0 (0.0)			
**IM**	0 (0.0)	15 (88.2%)	2 (11.8%)	0 (0.0)	*d vs*. *a*	0.000032	0.000023
**LGD**	2 (50%)	1 (25%)	1 (25%)	0 (0.0)	*d vs*. *b*	0.0050	0.012
**HGD**	0 (0.0)	6 (85.7%)	1 (14.3%)	0 (0.0)	*e vs*. *b*	0.082	1.00
**ACa *in situ***	0 (0.0)	4 (57.1%)	3 (42.9%)	0 (0.0)	*e vs*. *b*	0.088	0.13
**WD-ACa**	0 (0.0)	3 (50%)	3 (50%)	0 (0.0)	*e vs*. *b*	0.051	0.089
**MD-ACa**	0 (0.0)	3 (42.9%)	4 (57.1%)	0 (0.0)	*e vs*. *b*	0.020	0.038
**PD-Aca**	0 (0.0)	8 (61.5%)	3 (23.1%)	2 (15.3%)	*f vs*. *b*	0.14	0.18

Differentiation grade-specific subgroup profiles include *a*: Normal and *b*: IM. Testing subgroup profiles include *d*: Nuc, Nuc+Cyt, Cyt; *e*: Nuc+Cyt, Cyt; and *f*: Nuc+Cyt, Cyt, Neg. *p* < 0.05 is considered statistically significant.

**Fig 4:**
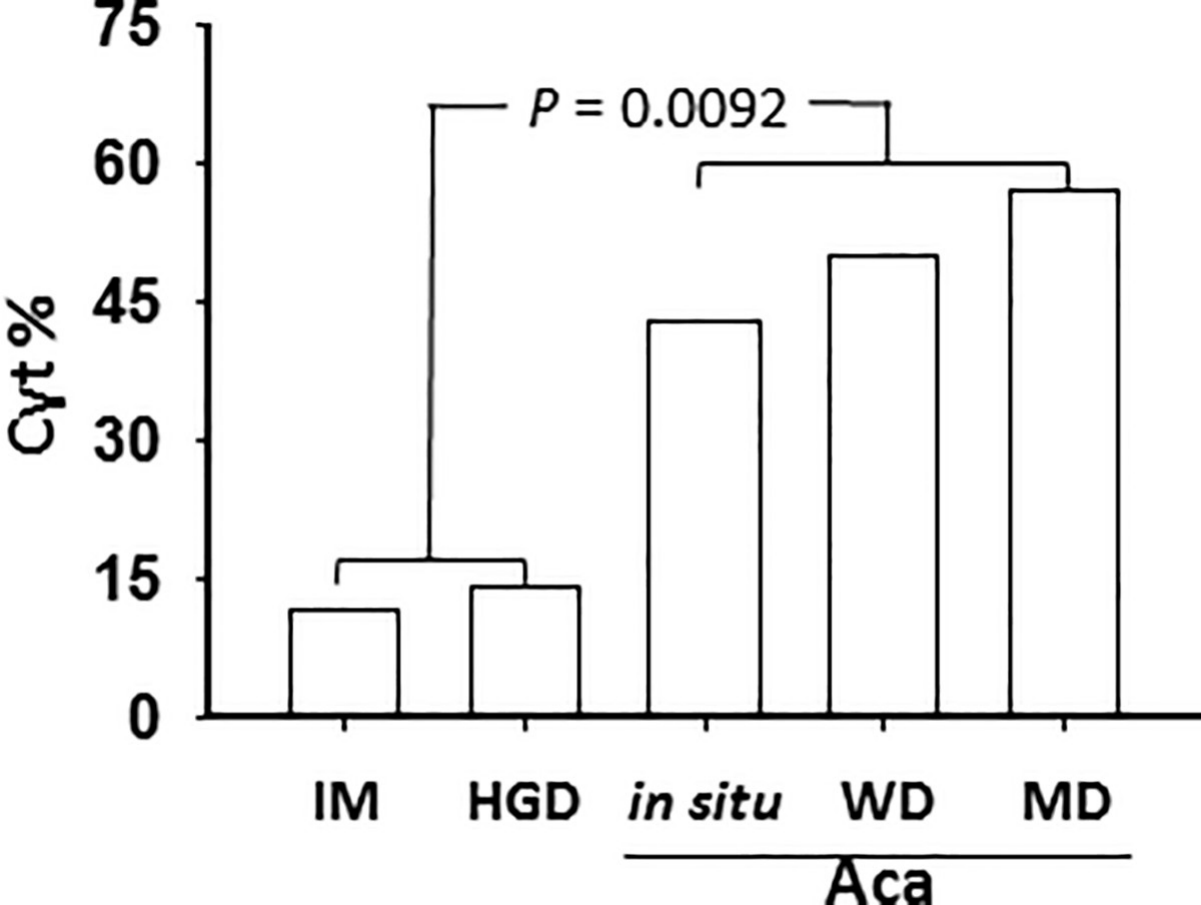
Maspin Cyt subtype correlates with ECA/GEJ Aca progression. Analysis of the percentage of Cyt subtype in each GEJ pathological grade. A statistically significant difference was found between the combined IM and HGD subtypes and the ECA/GEJ Aca subtypes (Fisher test: *p* = 0.0092).

MD-ECA/GEJ Aca, where the maspin Cyt pattern subtype became the majority, was significantly different from IM in subtype composition (χ^2^ test: *p* = 0.02; Fisher test: *p* = 0.038). Consistent with the progressively increased partition of the Cyt subtype from HGD to ECA/GEJ Aca *in situ*, to WD-ECA/GEJ Aca to MD-ECA/GEJ Aca (**Fig 3**), the *p* values in both the χ^2^ test and the Fisher exact test (*vs*. IM) progressively decreased. To examine how the partition of the Cyt subtype correlated with ECA/GEJ Aca progression we plotted the Cyt subtype percentages versus pathological grades, shown in **Fig 4**. In this analysis, the data points of LGD and PD-ECA/GEJ Aca were outliers since they were discordant from other GEJ lesions in terms of subtype composition. Analysis of the combined IM and HGD Cyt subtypes versus the combined *in situ*, WD- and MD-ECA/GEJ Aca subtypes revealed a statistically significant dramatic transition between the two groups (χ^2^ test: *p* = 0.0066; Fisher test: *p* = 0.0092). Prior to the critical transition to ECA/GEJ Aca *in situ* the partition of the Cyt subtype was steadily low, but not lower than 11 ± 4%. Among the three differentiation grades of ECA/GEJ Aca (i.e., ECA/GEJ Aca *in situ*, WD-ECA/GEJ Aca and MD-ECA/GEJ Aca), the partition of the Cyt subtype remained high, approaching a plateau of 54 ± 4%. These data thus suggest that the increase of the partition of the Cyt subtype coincided with the initiation of ECA/GEJ Aca.

### The differential expression of maspin does not overlap with IHC detection of TP53 in ECA/GEJ Aca progression

**Fig 5:**
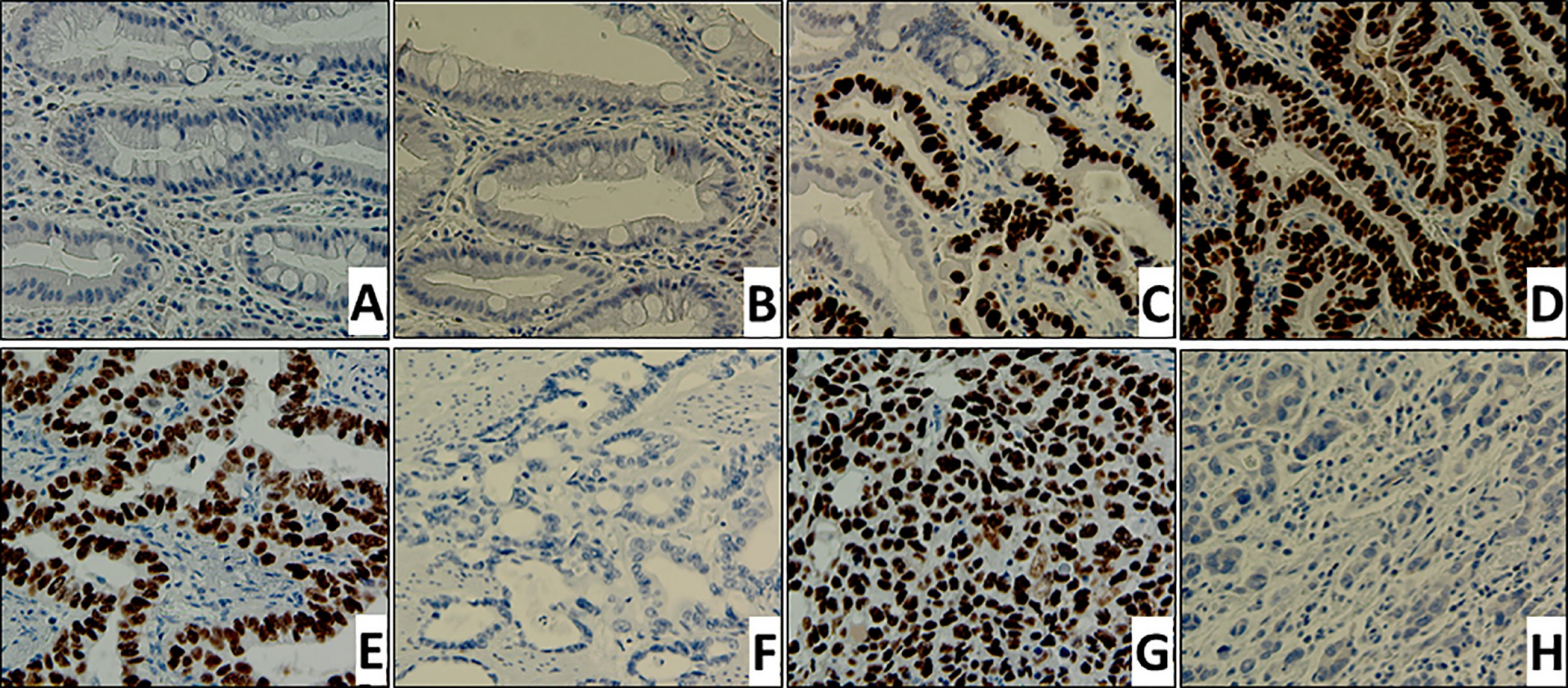
Differential patterns of TP53 IHC staining in ECA/GEJ Aca progression. Representative images of IHC staining (200x) of various GEJ lesions. **A.** and **B.** IM, **C**. LGD, **D**. HGD, WD-ECA/GEJ Aca to MD-ECA/GEJ Aca (**E** and **F**), and PD-ECA/GEJ Aca (**G** and **H**).

ECA/GEJ Aca is characterized by high inter-tumoral gene mutation heterogeneity. In particular, *TP53* deletion or mutation (signified by increased TP53 stability and IHC signal) occurs frequently in advanced ECA/GEJ Aca [40]. Thus, TP53 IHC detection is an unreliable marker of ECA/GEJ Aca, especially for early stages of disease progression. To assess whether the maspin expression pattern and TP53 IHC detection were concordant in ECA/GEJ Aca progression, we performed IHC of TP53 in parallel. **Fig 5** shows representative TP53 IHC results in ECA/GEJ Aca lesions of different histopathological grades. Consistent with earlier reports [15], normal mucosa and IM showed negative TP53 staining (**Fig 5A and 5B**) while LGD showed scattered TP53 nuclear staining (**Fig 5C**). Focal areas of HGD also showed TP53 nuclear staining (**Fig 5D**). Established ECA/GEJ Aca were either TP53 positive (**Fig 5E–5G**) and TP53 negative (**Fig 5F–5H**) whether the tumor was WD-, MD- (**Fig 5E and 5F**) or PD-ECA/GEJ Aca (**Fig 5G and 5H**). Our data are consistent with the earlier report that the genomic alterations of *TP53* often occur after the development of ECA/GEJ Aca [16], while the tumors that exhibited no TP53 staining may have undergone *TP53* gene deletion. Based on this result, the differential expression patterns of maspin were not concordant with those of TP53 in ECA/GEJ Aca. Thus, the potential clinical utility of maspin as a molecular marker for ECA/GEJ Aca early diagnosis is independent of TP53.

## Discussion

Taking advantage of the full spectrum of pathological changes from normal to invasive poorly differentiated adenocarcinoma in our tissue specimen collection, we provide here the first evidence that epithelial-specific maspin is differentially expressed in the hypothetical sequence of esophageal adenocarcinoma progression. The total level of maspin expression was progressively downregulated from normal GEJ epithelium to MD-ECA/GEJ Aca. Maspin was not detected in a subtype of invasive PD-ECA/GEJ Aca, further consistent with the accumulated evidence that maspin silencing may specifically contribute to the transition of tumor cells to invasive and metastatic phenotypes [22,24,25]. In light of the consensus that the maspin gene is not frequently mutated or deleted in cancer, the level of maspin expression is likely regulated at the level of transcription [41]. Importantly, GEJ lesions of each pathological grade could be divided into subtypes based on maspin subcellular distribution patterns. The progressive increase in the partition of the Cyt subtype revealed a statistically significant dramatic increase overlapping with the transition from HGD to ECA/GEJ Aca *in situ*.

Protein expression patterns can be used for cell lineage tracking in development and pathological processes [42]. Since the most dramatic increase of Cyt subtype occurred in the transition from HGD to ECA/GEJ Aca *in situ*, the Cyt pattern of maspin expression may be a particularly sensitive diagnostic marker of early stage ECA/GEJ Aca. Specifically, the Cyt subtype of pre-neoplastic GEJ lesions (i.e. IM and HGD) may be associated with a higher risk to progress to the Cyt subtype of ECA/GEJ Aca, and consequently may need to be closely monitored. Based on our regression analysis, tracking the Cyt subtype of IM or HGD of BE may lead to early diagnosis of approximately 50% of ECA/GEJ Aca (**Fig 4**). Consistently, we have shown that the tumor suppressive effect of maspin is reduced when it is excluded from the nucleus [36–38,43].

Increased Cyt maspin may be causative in ECA/GEJ Aca development and progression since the molecular mechanism underlying the tumor suppressive effect of maspin is, at least in part, through its direct inhibition of nuclear HDAC1 and HDAC1-dependent transcriptome [28,44]. It is conceivable that reduced inhibition of HDAC1, in the absence of nuclear maspin, may alter the chromatin folding and ultimately lead to genetic instability and DNA mutations. Thus, the differential expression of maspin, both at the level of total expression and subcellular distribution, may precede mutations of other tumor suppressor genes such as *TP53*. As shown in our study, the differential expression of maspin started at early stages of ECA/GEJ Aca development, whereas *TP53* mutation was detected in tumor cells of higher pathological grade. On the other hand, retention of maspin in the cytoplasm may contribute to increased tumor cell survival. We have shown that maspin localized in the cytoplasm interacts with glutathione-S-transferase (GST) [45,46] and 78 kDa glucose-regulated protein (GRP78) [44]. Consistent with the functions of GST and GRP78 in cellular defense against stress, cell lines of prostate, breast and lung cancers that express maspin in the cytoplasm demonstrate a significant survival advantage compared to those cells that do not express maspin in the mammosphere assay [27].

Further mechanistic studies are warranted to delineate whether and how the Cyt subtype of maspin expression pattern underlies the critical transition of HGD to ECA/GEJ Aca. It is worth noting that among benign and pre-neoplastic lesions (IM, LGD, and HGD), LGD was the only lesion that included a Nuc subtype. Currently, a school of thought recommends monitoring BE patients after LGD diagnosis. However, others have noted that LGD may result from other injuries rather than being a precursor of ECA/GEJ Aca [47–49], and cautioned that cancer screening in patients with LGD may lead to substantial unnecessary suffering of the patients at lower risk of developing cancer. To clarify this issue, we need to establish experimental models that recapitulate the cellular origin and progression of ECA/GEJ Aca. For example, evidence derived from a mouse model suggests that p63^+^KRT5^+^KRT7^+^ basal progenitor cells may be the progenitors for multi-layered epithelium and BE [21]. As biological and mechanistic insights are needed to validate this model, comprehensive profiling of gene expression combined with maspin expression patterns in tissue specimens may determine the clinically relevant connections among different lesions involved in ECA/GEJ Aca progression.

Overall, the results from the current study are highly significant for studying the underlying molecular mechanisms of ECA/GEJ Aca, and lend support to an exciting possibility of using the patterns of maspin subcellular distribution for cancer diagnosis and prognosis. Endoscopic imaging and biopsy pathology are currently used to monitor BE patients and diagnose ECA/GEJ Aca. This procedure is invasive and costly [3]. Considering that the rate of transition from pre-neoplastic lesions to carcinoma is low, it is intriguing to postulate that the Cyt type of maspin expression pattern in pre-neoplastic lesions may help assess cancer risk and help narrow the focus of cancer screening down to significantly smaller patient populations. To this end, temporal prospective or retrospective studies with larger cohorts are needed to determine how maspin expression patterns can sensitively and accurately stratify patients at risk of developing ECA/GEJ Aca. As a precautionary note, although the Nuc+Cyt subtype is progressively decreased in ECA/GEJ Aca progression, the evidence that both the Cyt subtype and the Nuc+Cyt subtype persisted in the full spectrum of ECA/GEJ Aca differentiation grade underscores the need for additional molecular markers that may serve as cancer risk factors within the Nuc+Cyt subtype.
